# Posterior interosseous artery perforator propeller flap for the repair of wrist and hand dorsal wounds

**DOI:** 10.3389/fsurg.2026.1786011

**Published:** 2026-02-24

**Authors:** Jian Han, Biyun Tang, Xiaojun Xie, Bai Lv, Yang Li, Kai Bin, Tian Xiang Ye, Yongfeng Su, Jianwen Cheng

**Affiliations:** 1 Trauma Orthopaedic Hand Surgery, The First Affiliated Hospital of Guangxi Medical University, Guangxi, Nanning, China; 2 Orthopaedic Ward II, The Second Affiliated Hospital of Guilin Medical University, Guangxi, Guilin, China; 3 Blood Transfusion Department, The Second Affiliated Hospital of Guilin Medical University, Guangxi, Guilin, China

**Keywords:** dorsal hand, dorsal wrist, perforator propeller flap, posterior interosseous artery, soft tissue defect

## Abstract

**Objective:**

This study aimed to evaluate the clinical efficacy of the posterior interosseous artery (PIA) perforator propeller flap for the repair of dorsal wrist and hand wounds.

**Methods:**

From October 2018 to October 2022, 12 patients with dorsal defects of the wrist (*n* = 5) or hand (*n* = 7) underwent repair using the PIA perforator flap. The surgical technique involved preoperative Doppler ultrasound localization of perforators, flap design centered on the selected perforator, and rotation of the flap up to 180° to cover the defect. Surgical outcomes were assessed based on flap survival, complications, and changes in preoperative/postoperative visual analog scale scores and disability of the arm, shoulder, and hand (DASH) scores.

**Results:**

Twelve cases of skin flaps were successfully harvested, and the donor sites were directly sutured. Postoperatively, 11 flaps survived completely, while one case developed a wound infection that resolved with debridement and dressing changes. Patients were followed for 6–18 months (mean 12 months). Flap texture and appearance were satisfactory, with no significant swelling observed. Patient satisfaction reached 91.67%. At the final follow-up, DASH scores ranged from 2 to 15, with a mean of 9.2. Mild scar hyperplasia was observed at the donor site in two cases and at the flap margin in one case.

**Conclusion:**

For repairing soft tissue defects of the dorsal wrist, the posterior interosseous artery perforator flap provides a valuable and reliable clinical option due to its consistent vascular supply, preservation of major vessels, minimal surgical complexity, favorable aesthetic and tactile qualities, minimal donor-site trauma, and high flap survival rate.

## 1 Introduction

The soft tissues of the dorsal wrist and hand are relatively thick and include deep structures such as extensor tendons. Inadequate management of traumatic soft tissue defects in this area can lead to severe complications, including tendon adhesions, non-union, and chronic infection. Repair of these defects requires exceptionally high standards for both functional recovery and cosmetic outcomes, thereby presenting a common clinical challenge (1, 2). Current repair methods primarily include free flaps and pedicled flaps. Free flaps, such as the radial artery superficial palmar branch flap or ulnar artery perforator flap, have yielded favorable outcomes. However, they require advanced microsurgical skills and are associated with significant surgical risks, which may limit their feasibility in resource-limited hospitals (3–6). Pedicled flaps, such as the traditional retrograde interosseous posterior artery island flap, provide a reliable blood supply and facilitate simpler surgical handling (7–11). However, maintaining a large pedicle to preserve vascular integrity frequently leads to complications such as postoperative swelling of the pedicle, substantial damage at the donor site, and inefficient tissue utilization (3, 9).

To overcome these limitations, researchers developed the perforator propeller flap technique. Hyakusoku et al. (4) first introduced this technique in 1991, and subsequent studies have further refined and advanced it (5, 6). The core principle involves high-angle rotational transplantation centered on a single perforator vessel, which facilitates precise point-to-surface reconstruction. This approach allows the larger paddle to effectively cover the defect, while the smaller paddle closes the donor site with minimal effort. As a result, tissue damage is minimized, and pedicle bulging is avoided. The versatility of this technique has been demonstrated in various anatomical regions. For instance, Chaudhuri et al. (3) reported favorable outcomes in treating distal lower limb defects, while Dagdelen et al. (12) achieved similarly positive results in elbow reconstruction. However, systematic research remains limited regarding the application of this advanced technique in combination with the reliable vascular supply and straightforward harvest of the posterior interosseous artery (PIA) perforator system, specifically for repairing dorsal wrist and hand defects. Although the PIA flap has been adapted into various configurations, including the antegrade flap for elbow reconstruction (13), the septal perforator-based flap (14), and other modifications (15), comprehensive analyses of the PIA perforator propeller flap for the repair of dorsal hand defects remain scarce. Therefore, this retrospective study aims to systematically evaluate the clinical efficacy of the PIA perforator propeller flap in the reconstruction of dorsal hand and wrist defects, thereby providing robust evidence to support its application.

## 2 Materials and methods

### 2.1 Study design and patient selection

We included 12 patients who underwent reconstructive surgery at our hospital between October 2018 and October 2022. All patients underwent interosseous posterior artery perforator propeller flap reconstruction for dorsal wrist or hand defects. The inclusion criteria are as follows: (1) age ≥18 years; (2) soft tissue defect located on the dorsal aspect of the wrist or hand; (3) inability to achieve direct suturing or free skin graft repair; and (4) treatment with the posterior interosseous artery perforator propeller flap. The exclusion criteria are as follows: (1) soft tissue defects not located on the dorsal aspect of the wrist or hand; (2) presence of severe systemic disease; and (3) poor compliance, with inability to cooperate with postoperative management following posterior interosseous artery perforator spiral-shaped flap treatment.

### 2.2 Surgical methods

The pedicle vessels were dissected along the main trunk of the PIA and vein or toward the anastomotic branch of the anterior interosseous artery as needed. The tourniquets were then released to observe the blood supply of the flap, ensuring that active bleeding was visible at the flap margins. After reperfusing the PIA and vein, the flap was rotated over the defect while carefully avoiding torsion, compression, or entrapment of the perforating vessels. The flap periphery and donor-site skin were secured with sutures, confirming active bleeding at the flap margins. Finally, once adequate flap perfusion was established, the defect was closed and soft sterile dressings were applied.

### 2.3 Postoperative management and follow-up

Postoperatively, radiant heat lamps were applied to maintain limb warmth at approximately 26°C. The wrist was immobilized in a functional position using a plaster cast for 1 week to reduce flap tension. Routine antimicrobial therapy was administered for 3 days. Flap parameters, including skin color, temperature, tension, and capillary refill responses, were closely monitored and documented. Except for activities such as eating and toileting (which could be performed while standing or sitting), patients were instructed to remain on bed rest for 1 week. If signs of venous return impairment, such as hematoma formation or excessive flap tension, were observed, the medical team performed partial suture removal or flap massage as appropriate. In cases of partial necrosis, surgical debridement or regular dressing changes were performed according to the severity of the condition. Sutures and casts were removed at 3 weeks postoperatively, after which wrist joint functional exercises were gradually initiated. Follow-up appointments were scheduled at 1, 3, 6, and 12 months postoperatively, with subsequent visits arranged as needed. Functional outcomes were objectively assessed at 1 month and the final follow-up using the disability of the arm, shoulder, and hand (DASH) questionnaire. Pain intensity was evaluated using the visual analog scale (VAS). At the final follow-up, patient satisfaction with cosmetic and functional outcomes was assessed using a simple questionnaire. Patients rated their overall satisfaction as “very satisfied,” “satisfied,” “neutral,” “dissatisfied,” or “very dissatisfied.” Patient satisfaction was calculated as the percentage of patients who reported “satisfied” or “very satisfied.”

### 2.4 Statistical analysis

We used GraphPad Prism 8.0 software for all statistical analyses. Data in this study are expressed as mean ± standard deviation (SD). The Wilcoxon signed-rank test was used to compare preoperative and postoperative outcomes.

## 3 Results

### 3.1 Patient injury characteristics

We included 12 patients who underwent reconstructive surgery at our hospital between October 2018 and October 2022. All patients underwent interosseous posterior artery perforator propeller flap reconstruction for dorsal wrist or hand defects. Among them, eight were men and four were women, with ages ranging from 20 to 65 years (mean: 37.1 years). The etiology of injury included crush injury (five cases), laceration (three cases), strangulation (two cases), and infection (two cases). Soft tissue defects were located on the dorsal wrist (five cases) or dorsal hand (seven cases), and all cases involved exposed tendons or bone. Wound defect sizes ranged from 5 cm × 4 cm to 3 cm × 3 cm. Associated injuries included metacarpal fractures (five cases) and tendon ruptures (four cases). The injury characteristics of the included patients are presented in Table 1. Primary management consisted of vacuum sealing drainage (VSD) for wound protection and infection control, followed by secondary flap repair.

**Table 1:** Patient demographics and injury characteristics (*n* = 12).

Case no.	Sex/age (years)	Etiology	Defect location	Defect size (cm)	Comorbid injuries
1	M/34	Crush	Hand dorsum	4.0 × 3.0	–
2	F/29	Laceration	Wrist dorsum	3.5 × 3.0	–
3	M/21	Avulsion	Hand dorsum	5.0 × 4.0	Tendon rupture
4	M/51	Infection	Wrist dorsum	4.0 × 3.5	–
5	F/61	Crush	Hand dorsum	4.5 × 4.0	–
6	M/23	Laceration	Hand dorsum	3.5 × 3.0	Tendon rupture
7	M/38	Avulsion	Wrist dorsum	4.0 × 3.0	–
8	F/47	Infection	Hand dorsum	5.0 × 4.0	–
9	M/56	Crush	Hand dorsum	5.0 × 4.5	Tendon rupture
10	F/65	Infection	Wrist dorsum	4.0 × 3.0	Tendon rupture
11	M/20	Laceration	Wrist dorsum	3.0 × 3.0	–
12	M/41	Crush	Hand dorsum	5.0 × 4.5	–

M, male; F, female.

### 3.2 Surgical and functional outcomes

**Figure 1:**
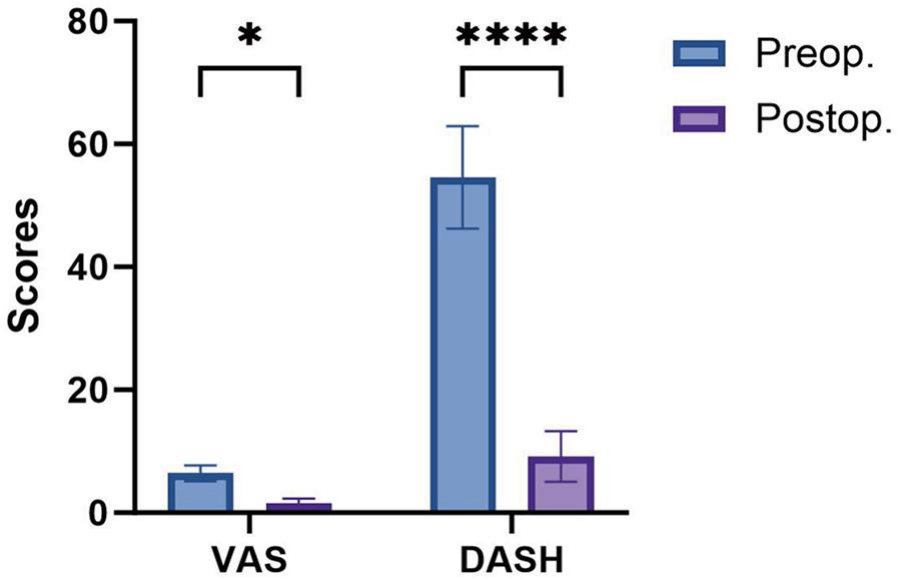
Comparison of preoperative and 1-month postoperative VAS and DASH scores. Data are presented as mean ± SD. *p < 0.05, ****p < 0.0001.

All flap procedures were performed successfully. The PIA was consistently identified, with 3–4 cutaneous perforators observed in the middle and lower thirds of the forearm. Graft dimensions ranged from 18 cm × 5 cm to 12 cm × 3.5 cm, and all defects were completely covered. Donor sites were closed using direct suturing. One patient experienced distal edge necrosis, and another developed perilesional wound infection; however, both cases healed progressively after debridement and dressing changes. All other flaps survived successfully. Patients were followed up for 6–18 months, with a mean follow-up of 12 months. All flaps exhibited good texture and appearance, with no significant swelling observed. The final DASH scores ranged from 2 to 15 (mean: 9.2), and the final VAS scores ranged from 0 to 3 (mean: 1.5) (Tables 2, 3). Compared to preoperative values at 1 month, both VAS and DASH scores showed significant reductions at 1 month postoperatively (Figure 1 and Table 4). Mild scar hyperplasia was observed at the donor sites in two cases and at the flap margin in one case.

**Table 2:** Surgical details and postoperative outcomes.

Case no.	Flap size (cm)	Combined injuries	Postoperative complications	Postop. DASH	Postop. VAS
1	12.0 × 3.5	–	–	5	1
2	12.0 × 4.0	–	–	8	0
3	16.0 × 3.5	Tendon rupture	–	12	2
4	12.0 × 3.5	–	Wound infection	7	1
5	13.0 × 4.0	–	–	15	2
6	15.0 × 5.0	Tendon rupture	–	4	1
7	12.5 × 3.5	–	–	9	1
8	13.0 × 4.0	–	–	11	2
9	18.0 × 5.0	Tendon rupture	Distal marginal necrosis	14	2
10	14.0 × 4.0	Tendon rupture	–	10	2
11	12.5 × 3.5	–	–	2	1
12	16.5 × 4.5	–	–	13	3

**Table 3:** Preoperative and 1-month postoperative VAS and DASH scores for all patients.

Case no.	Preop. VAS	Postop. VAS	Preop. DASH	Postop. DASH
1	7	1	52	5
2	6	0	48	8
3	8	2	65	12
4	5	1	45	7
5	7	2	58	15
6	6	1	50	4
7	7	1	55	9
8	6	2	60	11
9	8	2	68	14
10	5	2	52	10
11	4	1	40	2
12	8	3	62	13

**Table 4:** Comparison of preoperative and postoperative VAS and DASH scores.

Parameter	Preoperative (mean ± SD)	Postoperative (mean ± SD)	*p*-Value
VAS score	6.3 ± 1.3	1.4 ± 0.8	0.002*
Dash score	54.3 ± 8.3	9.2 ± 4.1	0.002*

*p*-Values were calculated using the Wilcoxon signed-rank test. Significance levels are indicated as follows.

* *p* < 0.01.

### 3.3 A typical case patient

**Figure 2:**
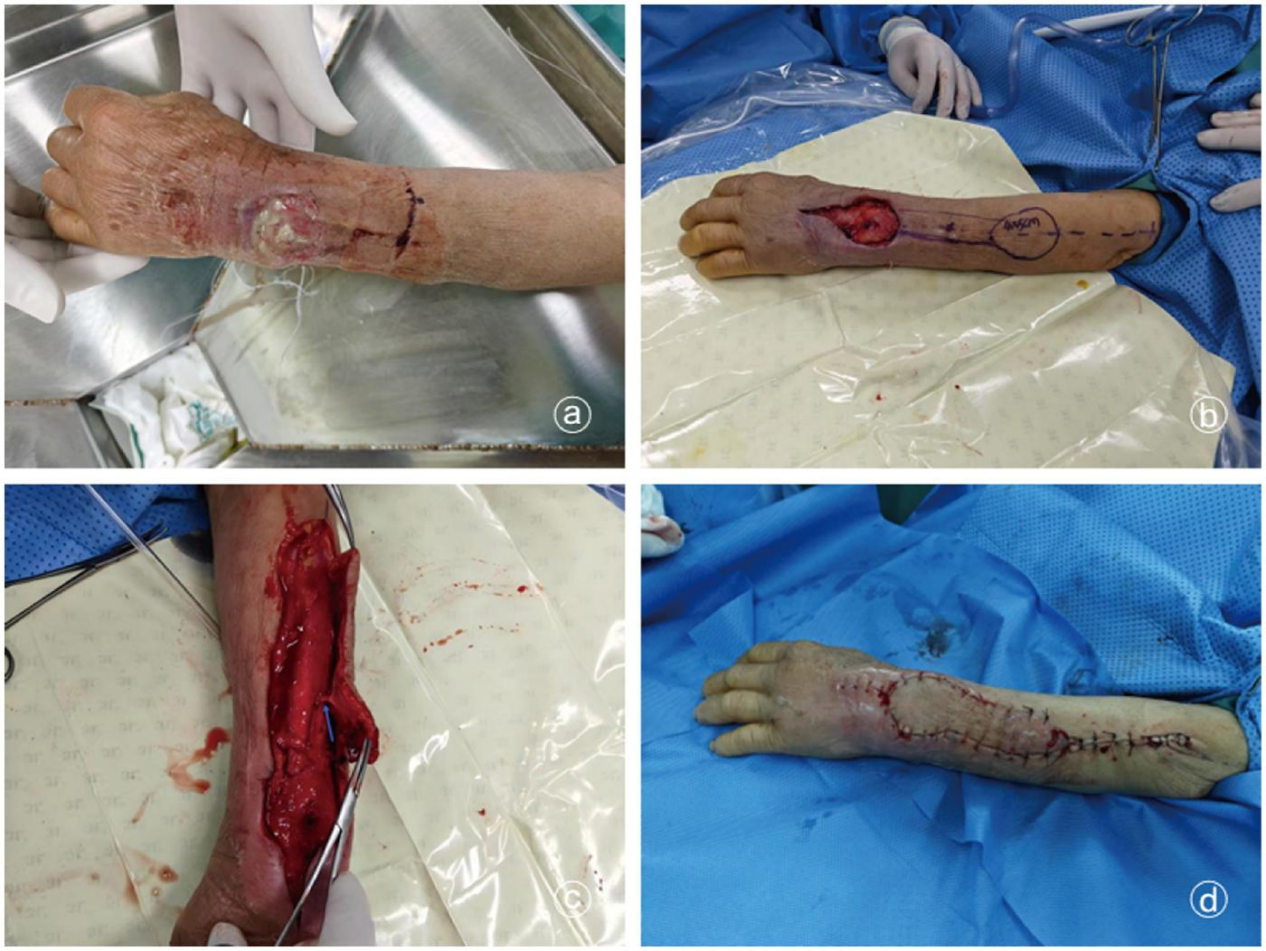
Wound condition before and after treatment. **(a)** On admission, an infected wound on the dorsal wrist was observed, with purulent exudate formation and exposed tendons. **(b)** After thorough debridement, the wound was protected with a VSD to control inflammation and eliminate infection. The wound defect measured 4 cm × 3 cm. A PIA propeller flap transfer was planned, with the repair flap measuring 5 cm × 4 cm. **(c)** Intraoperative flap mobilization revealed only perforating vessels, indicated by the arrow, which remained connected to the flap. **(d)** The procedure proceeded smoothly, with good flap perfusion observed postoperatively.

**Figure 3:**
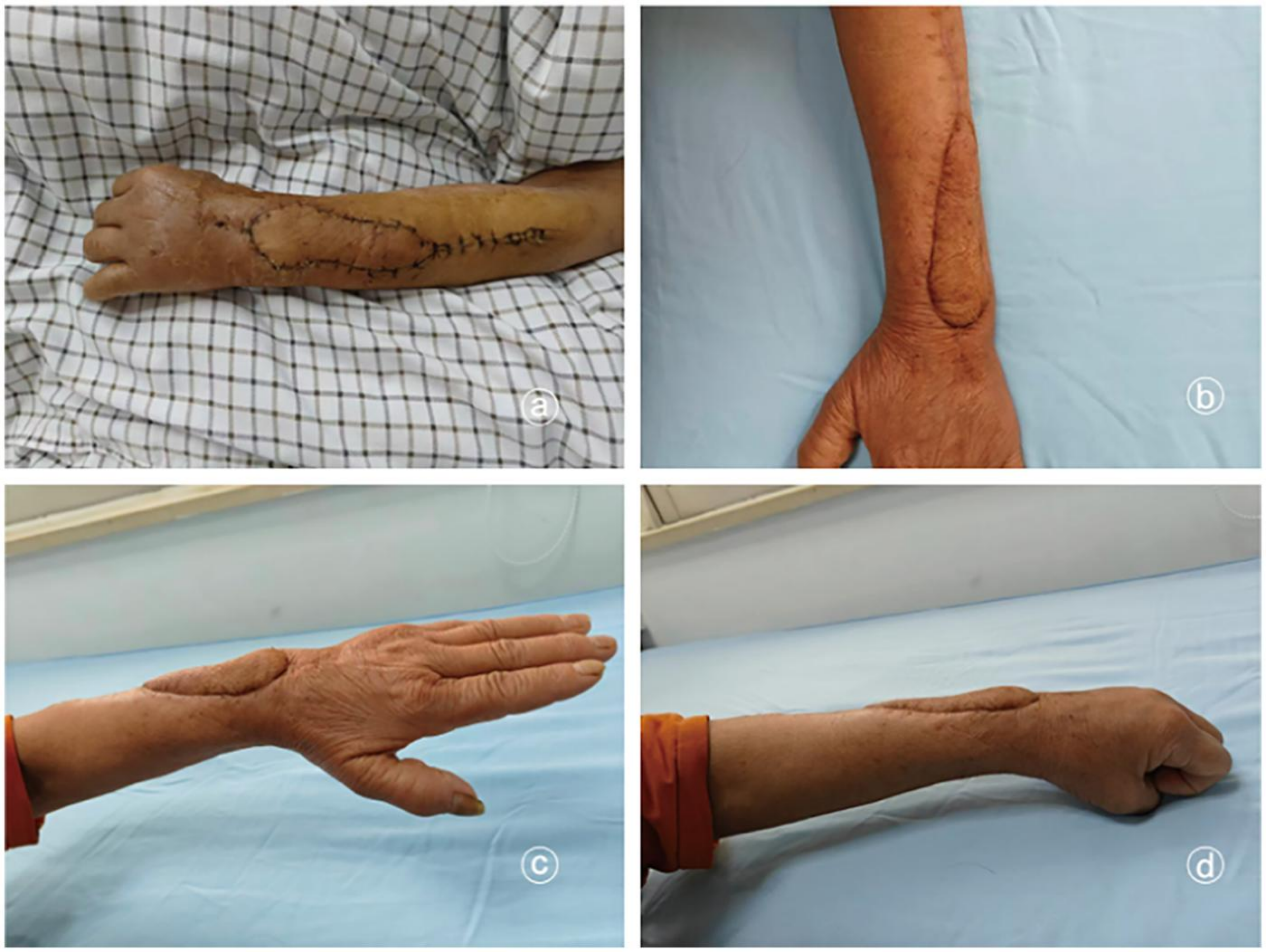
Detection of the appearance and wrist and hand function after flap repair after operation. **(a)** One week after operation, the flap showed smooth survival; **(b)** at 8 weeks postoperation, the appearance and texture of the flap had significantly improved; **(c)** at 8 weeks after operation, the extension function of the left wrist joint and fingers was well-restored; and **(d)** at 8 weeks after operation, the flexion function of the left wrist joint and fingers demonstrated satisfactory recovery.

A typical case involved a 65-year-old woman who was admitted with redness, swelling, and exudate on the dorsal aspect of her left wrist following trauma (Figure 2). After thorough debridement, the wound was covered with a VSD dressing. Once the inflammation subsided, a residual defect measuring approximately 4 cm × 3 cm remained. An interosseous posterior artery propeller flap transfer was planned, and the primary flap was designed to measure 5 cm × 4 cm. During surgery, the flap was anatomically dissected, preserving only the perforating vessels connected to it. The procedure proceeded smoothly, with satisfactory postoperative flap perfusion. Consequently, flap survival was confirmed at 1 week postoperatively. At 8 weeks postoperatively, the flap exhibited a favorable appearance and texture. Flexion and extension functions of the left wrist joint and fingers returned to normal (Figure 3).

## 4 Discussion

### 4.1 Anatomical basis of PIA

The PIA originates from the common interosseous artery posterior to the proximal forearm. It descends behind the interosseous membrane of the forearm and forms communicating branches with the anterior interosseous artery in the distal forearm. In 1988, Zancolli and Angrigiani (16) first reported a PIA flap. In their study, they divided the PIA into three segments and described its path and branches, noting that multiple perforators arise along the course of the PIA. Three types of perforators have been identified: (1) Type 1 consists of distal and proximal vascular plexuses, each containing 3–4 perforators; (2) Type 2 includes multiple perforators distributed along the PIA, spaced 1–2 cm apart; and (3) Type 3 features a single thick perforator originating proximally, homologous to the recurrent branch of the PIA. This perforator has a considerable diameter and bifurcates into several branches, averaging six in number. This anatomical classification provides theoretical guidance for the precise localization and dissection of the PIA flap.

### 4.2 Relationship between the PIA and the posterior interosseous nerve

PIA is closely associated with the posterior interosseous nerve (PIN). Keogh et al. (17) provided a detailed description of the branches of the PIN but did not describe the accompanying relationship between the PIA and the PIN. Angus et al. (18) described the close relationship between the PIA and nerve through the analysis of 10 autopsies and concluded that the anatomy of the PIA flap can be divided into three regions, with the risk of nerve injury gradually decreasing from proximal to distal dissection. During dissection of the nerve and artery, safe harvesting of the flap can be achieved through careful preservation of the fine motor branches of the PIN. This finding provides guidance for better protection of the PIN during PIA flap harvesting. In our study, all 12 perforators were located in the mid-to-distal lower-risk regions of the PIA, and no injuries to the muscular branches of the PIN were observed. Postoperative recovery of finger extension function was favorable, confirming this theory. Given the close relationship between the PIA and nerve, using a head-mounted surgical loupe or microscope during dissection of perforating vessels is safer (19).

### 4.3 Advantages and disadvantages of the traditional retrograde PIA flap

The retrograde PIA flap is one of the commonly used techniques for repairing the dorsal wrist–metacarpal joint, web space, and surrounding areas (12, 20). It is widely applied primarily through two approaches: the fasciocutaneous pedicle flap and the axial flap. The main advantage of the fasciocutaneous flap is its relatively straightforward harvesting. The proximal end does not require deep dissection of vessels or nerves, thereby minimizing the risk of nerve injury. It can also incorporate muscle and tendon to form a composite flap (21). The disadvantage is that, to ensure adequate blood supply, the pedicle is typically wide, which may result in extensive tissue damage during harvest, noticeable scarring, significant tissue waste, and a bulky, thick pedicle. The advantage of the axial flap is its ability to incorporate more perforating vessels, allowing for a lobulated design, and it can also be harvested as a composite flap, offering greater flexibility. However, its disadvantages include difficulty in freeing the proximal flap, a risk of injury to the posterior interosseous nerve, and the need to free muscle and tendon tissue, which may adversely affect finger extension function.

### 4.4 Advantages and disadvantages of the propeller flap

Professor Hyakusoku (4) first reported the perforator propeller flap in 1991, marking a landmark progress in the development of flap surgery. This technique offers several advantages, including convenient sampling, avoidance of microscopic dissection, good flap texture, excellent cosmetic outcomes, and minimal donor-site injuries. It therefore holds significant potential for the repair of dorsal wrist and hand wounds. The PIA perforator propeller flap offers the following advantages: (1) After the flap is harvested and rotated, the larger paddle is used to repair wounds of the wrist and dorsum of the hand, while the smaller paddle repairs part of the donor site. This method minimizes tissue waste, adheres to the economic principles of tissue transplantation, and causes less injury. (2) The donor site with a flap width of less than 4 cm can generally be directly sutured without the need for skin grafting, thereby avoiding scar hyperplasia that may occur after skin grafting. (3) The procedure is relatively simple because there is no need to separate the PIA and the PIN in the deep proximal flap. (4) Dissection in the middle and distal forearm basically does not require exposure of the radial nerve, effectively reducing the risk of extensor function injury. The disadvantages include (2) a relatively limited repair range, as the technique is only suitable for defects of the dorsal side of the wrist and hand, with limited application for defects in other areas; (2) a large incision on the forearm, which leaves more noticeable scars at the donor site and may affect the aesthetic outcomes and may be a concern for patients with high cosmetic or social expectations, particularly young women; and (3) the possibility of mislocation or accidental damage to perforator vessels supplying the flap, which can lead to surgical failure. As reported by Hamada et al. (22), the success of a propeller flap based on other forearm perforators relies heavily on the precise identification and preservation of these perforator vessels. The success of the procedure requires the surgeon to have a thorough understanding of flap anatomy and may benefit from the advanced preoperative imaging evaluations, as previously defined.

The PIA perforator propeller flap, a modification of the traditional PIA flap, represents an advanced surgical technique. In addition to focusing on critical aspects such as the pivot point, axial orientation, and surface dimension during flap elevation, surgeons should also consider the following factors: (1) preoperative use of Doppler ultrasonography along the surface projection of the PIA can help surgeons identify the perforator vessels closest to the wound. Color Doppler imaging is particularly advantageous due to its ability to provide detailed visualization of blood flow patterns. (2) After the start of surgery, an incision should be made between the extensor digiti minimi tendon and the extensor carpi ulnaris tendon to locate the effective perforator vessels closest to the wound to avoid injury. (3) The perforator pedicle should be carefully exposed, and the rotational flap should be evaluated intraoperatively. Based on the degree of vascular pedicle tension and flap blood supply, it is determined whether the main PIA vessels need to be freed to increase the vascular torsion distance and reduce the risk of venous crisis caused by compression (23–25). (4) Postoperative elevation of the affected limb, close observation of flap blood supply, and reasonable use of anticoagulants are essential to avoid a blood supply crisis. In this group, one patient developed necrosis in the distal part of the flap, which was considered to be caused by slight intraoperative entrapment that went undetected. Therefore, it is crucial to ensure that the perforator vessel pedicle is completely exposed during surgery.

Based on our experience, we emphasize several technical refinements to minimize vascular risks, particularly venous congestion caused by pedicle kinking. Meticulous subfascial dissection under loupe magnification is performed to preserve a sufficient length (typically 1.5–2 cm) of the perforator pedicle, which allows for a rotation of up to 180° without inducing tension. After flap elevation, a “trial rotation” is routinely conducted to ensure that the pedicle remains free from twisting or compression prior to final placement. In addition, the skin around the pedicle is sutured loosely, and a small suction drain may be placed away from the pedicle to avoid hematoma-related compression. These measures, combined with postoperative monitoring, contributed to the favorable outcomes observed in this series. Regarding feasible dimensions of the flap, our experience suggests that a propeller flap based on a single robust perforator located in the middle or distal third of the forearm can reliably cover defects up to 20 cm × 6–7 cm. In the present series, the largest flap harvested measured 18 cm × 5 cm and remained fully viable. Flap design must ultimately be individualized, guided by preoperative imaging and patient-specific factors.

In summary, the interosseous posterior artery perforator propeller flap offers numerous advantages for repairing soft tissue defects of the dorsal wrist. It provides a stable blood supply, causes minimal damage to major vessels, carries a low risk of nerve injury, and involves a straightforward surgical technique. In addition, this flap provides excellent cosmetic appearance and texture, causes minimal donor site trauma, and exhibits a high survival rate. These characteristics make it a safe and effective treatment option for such cases.

### 4.5 Limitations

This study is a retrospective case series and therefore has inherent limitations. First, its retrospective design may introduce potential selection bias. Second, the small sample size (*n* = 12) may limit the generalizability of the findings. Third, the lack of a control or comparison group may prevent direct comparative efficacy assessment. Future prospective studies with larger cohorts are warranted to further validate these results.

## 5 Conclusion

Our study has shown that the PIA perforator propeller flap is a feasible, reliable, and effective option for repairing soft tissue defects of the dorsal hand.

## Data Availability

The original contributions presented in the study are included in the article/Supplementary Material, further inquiries can be directed to the corresponding authors.
